# A Case of Gastric Glomus Tumor Misdiagnosed as Carcinoid Tumor

**DOI:** 10.7759/cureus.34316

**Published:** 2023-01-28

**Authors:** Kosisochukwu J Ezeh, William Boateng, Bidhan Paudel, Obiora Ezeudemba, Youssef Botros

**Affiliations:** 1 Internal Medicine, Jersey City Medical Center, Jersey City, USA; 2 Internal Medicine, St. Vincent's Medical Center, Bridgeport, USA; 3 Gastroenterology and Hepatology, Jersey City Medical Center, Jersey City, USA

**Keywords:** synaptophysin, mesenchymal stromal cells, gastrointestinal stromal tumor (gist), carcinoid tumour, gastric glomus tumors

## Abstract

Glomus tumor is a rare mesenchymal tumor commonly located in the periphery of glomus bodies, such as the subungual regions (e.g., fingernails and toenails). Other locations include the forearm, wrist, or trunk. Even rare is when these tumors are found in the submucosa. In the stomach, it is commonly found at the gastric antrum. Gastric glomus tumors (GGTs) are often found incidentally after a presumption of other gastric tumors is diagnosed, such as gastrointestinal stromal tumors (GISTs) or carcinoid tumors. The variable clinical presentation of GGT and the fact that histology is the only way to confirm the diagnosis is what makes GGT such an elusive tumor. Our case is a patient that presented with weight loss and reflux. Esophagogastroduodenoscopy (EGD) and colonoscopy were done, and the diagnosis of carcinoid tumor was presumed. Preliminary pathology was suggestive of a diagnosis of carcinoid tumor. The patient eventually had a subtotal gastrectomy, and a biopsy with immunohistochemical staining of the specimen was received, finally confirming the diagnosis of a GGT.

## Introduction

Glomus tumors are rare benign tumors of mesenchymal origin. They are usually located in the distal extremities and rarely seen in the GI tract, accounting for less than one percent of all GI soft tissue tumors [1]. Apart from peripheral soft tissues, other sites reported to be involved are the osteoarticular system, muscle, mediastinum, lung, kidney, and uterus. Given their location and appearance, glomus tumors are often mistaken for carcinoid tumors and other gastric subepithelial lesions, making their diagnosis dependent primarily on pathological and immunohistochemical findings [2-3]. Herein, we describe a case of gastric glomus tumor (GGT) initially misdiagnosed as a carcinoid tumor.

## Case presentation

**Figure 1:**
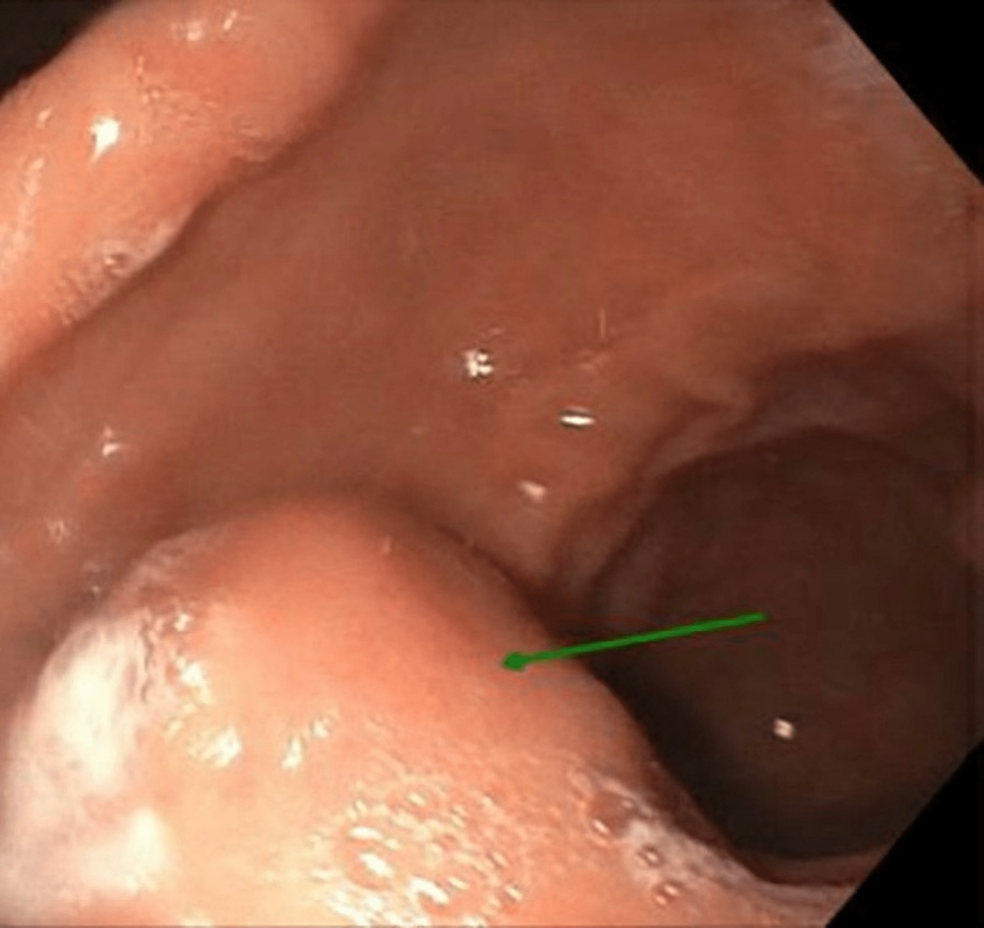
Esophagogastroduodenoscopy (EGD) finding of a large hiatal hernia, gastritis, and a submucosal lesion seen in the gastric antrum along the anterior wall. EGD - Esophagogastroduodenoscopy

We present a 67-year-old male with a history of hypertension, hyperlipidemia, membranous urethral stricture status post direct vision internal urethrotomy, and cerebrovascular accident, referred for an endoscopic ultrasound with biopsy for complaints of weight loss and reflux symptoms following an esophagogastroduodenoscopy (EGD). Findings of a large hiatal hernia, gastritis, and a submucosal lesion were seen in the gastric antrum along the anterior wall, as depicted in Figure 1. The patient’s reflux was responsive to proton pump inhibitors. The patient previously had an EGD and colonoscopy. EGD and colonoscopy showed one tubular adenoma and internal hemorrhoids. 

**Figure 2:**
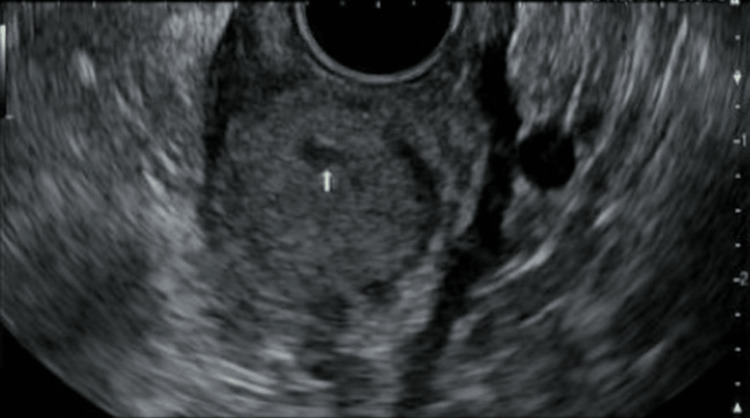
Endosonography (EUS) showing an 18.5 x 17.5 mm lesion in the gastric wall.

The patient stated that he had a good appetite without nausea, vomiting, or other lower GI symptoms. The patient denied smoking, alcohol, or illicit drug use. All other history was non-pertinent. CT scans of the abdomen with and without contrast showed mild thickening of the gastric wall without evidence of obstruction. Endosonography showed an 18.5 x 17.5 mm lesion in the gastric wall from the muscularis propria, slightly hypoechoic with a heterogenous appearance, multiple vessels within the lesion, small area of cystic degeneration, as depicted in Figure 2.

**Figure 3:**
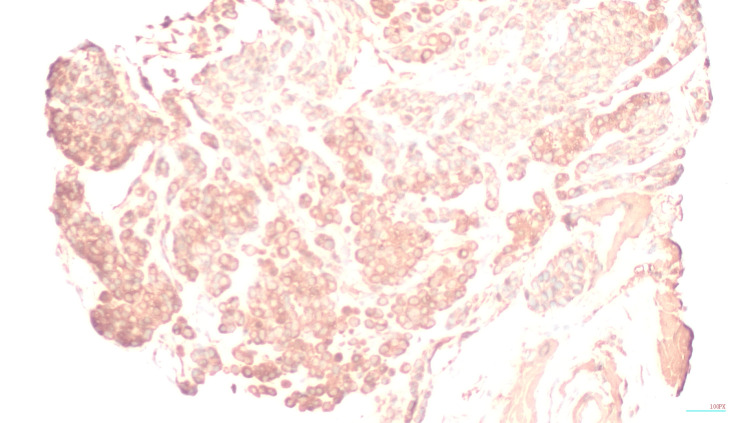
Immunohistochemical stain with smooth muscle actin (SMA). Tumor cells show a strong positive cytoplasmic stain for SMA.

**Figure 4:**
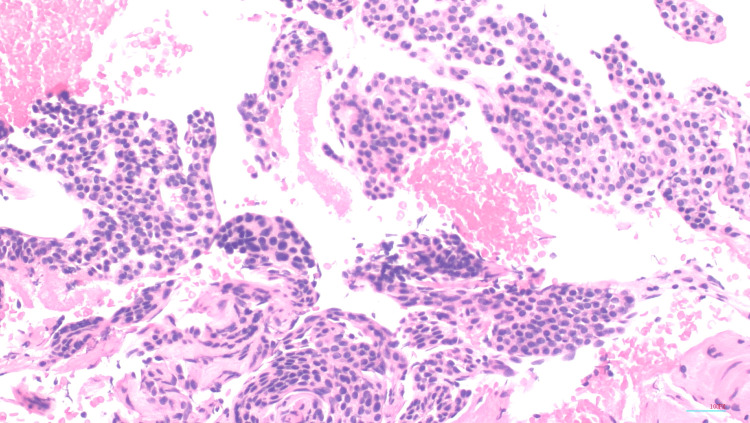
H&E histology stain. The tumor comprises nests, or solid sleets of uniform cells with round nuclei and scant pink cytoplasm. Nuclear atypical and mitotic figures are absent.

**Figure 5:**
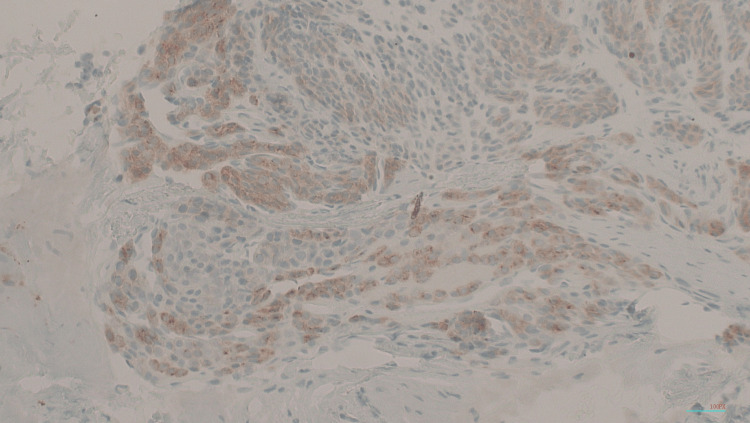
Immunohistochemical stain of synaptophysin. Tumor cells show moderate cytoplasmic stains for synaptophysin.

Fine needle aspiration biopsy showed features suggestive of a carcinoid tumor positive for synaptophysin (CD56 (-), chromogranin (-), synaptophysin (+), Ki67~2%). Synaptophysin is a neuroendocrine marker that is specific and fairly sensitive for neuroendocrine tumors [3]. Ki67 is an indication of the mitotic index or the level of cellular proliferation [4]. With these findings, a neuroendocrine tumor was confirmed. Type 3 gastric carcinoid tumor (T3GCT) is normally confirmed via normal gastrin levels and histologic characteristics showing poorly differentiated endocrine and exocrine cells extending into deep layers. T3GCT has the greatest potential for metastasis. The patient was counseled and scheduled for surgical resection. The patient had a subtotal gastrectomy of the distal portion of the stomach. The lesion was mildly hemorrhagic (pink-purple, polypoid, rubbery nodule along the serosa measuring 1.5 x 1.0 x 0.6 cm). Multiple biopsies were taken, and all margins and surrounding omentum were negative for tumor. They appear as round cells devoid of atypia. Surrounding LN were negative for malignant cells. Immunohistochemical stains showed chromogranin (-), synaptophysin (+), smooth muscle actin (SMA) (+), calponin (+), and Ki-67. GGT was the finalized diagnosis after histology showing positive SMA and calponin [5], as seen in Figures 3-5.

## Discussion

GGTs are rare entities. Since its first notation in 1928 [4], it has been increasingly acknowledged as a pathology that presents with varying symptoms such as epigastric pain, hematochezia, and in most cases, is asymptomatic, that is, discovered incidentally on endoscopy. They have a female predilection within the fifth and sixth decades [6]. When given the consideration of a tumor, a major concern is its malignant potential. Less than 1% have been reported [6], and they usually fulfill these criteria: large size greater than 2 cm, deep seated position, and high mitotic figures [7]. The case highlights two important phenomena. First is the rarity of GGTs, and second is the morphological overlap it possesses with more commonly encountered neuroendocrine tumors that lead to diagnostic pitfalls. GGT is seen as a polypoidal lesion with intact mucosa. Unfortunately, imaging studies such as CT and MRI cannot confirm the diagnosis or accurately differentiate GT from other submucosal tumors.

GGT is often misdiagnosed, as in our case with other more common submucosal tumors of mesenchymal origin, like gastrointestinal stromal tumors (GIST), carcinoid tumors, and schwannomas [8]. Its accurate diagnosis depends on both histopathology and immunohistochemical stains to confidently differentiate it from its counterparts. In our case, the preliminary diagnosis entertained was that of a neuroendocrine neoplasm. Immunohistochemical stains were positive for synaptophysin but negative for DOG-1 and chromogranin (a non-specific neuroendocrine marker). Further studies revealed that the tumor was positive for SMA and calponin. As a result, GGT was the finalized diagnosis. Synaptophysin is a neuroendocrine marker that is specific and fairly sensitive to neuroendocrine tumors [3]. Synaptophysin can be positive in about 20% of GGT cases, which may falsely result in these tumors being diagnosed as carcinoid tumors. Carcinoid/Neuroendocrine tumors are also positive for chromogranin, cytokeratin, CD56, and NSE, with consistent negative stains for SMA and CD34, which are positive in GT. CD34 can be focally positive in a few GGTs, while the peripheral GTs are more commonly positive. There are three types of gastric neuroendocrine tumors (NETs) depending on their production of the hormone gastrin. Type 1, the commonest variant, is usually benign in nature and small in size. It is noted to have high gastrin levels. Type 2, although rare, is mostly seen in people with genetic and hormonal conditions such as MEN1, and gastrin levels might be high. Type 3, usually the larger variant, is commonly seen in men over 50. The level of gastrin is not high [9]. Current treatment involves wedge resection with negative margins, which our patient underwent [10]. If it involves the pylorus, distal gastrectomy with reconstruction is considered to avoid gastric outlet obstruction. Other treatment options utilizing endoscopic methods like endoscopic submucosal dissection exists. In general, GGT has a good prognosis following achieving negative margins from resection, but there have been rare cases of metastases to distant sites. Thus, surveillance and close monitoring are required.

## Conclusions

This case demonstrates the elusiveness in diagnosing GGTs even after initial histology. Even with histology, certain positive stains may lead us in the wrong direction. Though glomus tumors are generally benign, there is potential for malignancy. It is essential to highlight the unique clinical presentations that GGTs present with. Despite how rare GGT is, it warrants being included in the differential diagnosis along with GIST, carcinoid, and lymphoma. It is ultimately important to definitively treat this tumor with resection. 
